# DNA Double-Strand Breaks Induce the Nuclear Actin Filaments Formation in Cumulus-Enclosed Oocytes but Not in Denuded Oocytes

**DOI:** 10.1371/journal.pone.0170308

**Published:** 2017-01-18

**Authors:** Ming-Hong Sun, Mo Yang, Feng-Yun Xie, Wei Wang, Lili Zhang, Wei Shen, Shen Yin, Jun-Yu Ma

**Affiliations:** 1 College of Animal Science and Technology, Key Laboratory of Animal Reproduction and Germplasm Enhancement in Universities of Shandong, Qingdao Agricultural University, Qingdao, China; 2 Center of Reproductive Medicine, Department of Obstetrics and Gynecology, Peking University Third Hospital, Beijing, China; 3 Institute of Reproductive Science, Qingdao Agricultural University, Qingdao, China; Institute of Zoology Chinese Academy of Sciences, CHINA

## Abstract

As a gamete, oocyte needs to maintain its genomic integrity and passes this haploid genome to the next generation. However, fully-grown mouse oocyte cannot respond to DNA double-strand breaks (DSBs) effectively and it is also unable to repair them before the meiosis resumption. To compensate for this disadvantage and control the DNA repair events, oocyte needs the cooperation with its surrounding cumulus cells. Recently, evidences have shown that nuclear actin filament formation plays roles in cellular DNA DSB repair. To explore whether these nuclear actin filaments are formed in the DNA-damaged oocytes, here, we labeled the filament actins in denuded oocytes (DOs) and cumulus-enclosed oocytes (CEOs). We observed that the nuclear actin filaments were formed only in the DNA-damaged CEOs, but not in DOs. Formation of actin filaments in the nucleus was an event downstream to the DNA damage response. Our data also showed that the removal of cumulus cells led to a reduction in the nuclear actin filaments in oocytes. Knocking down of the *Adcy1* gene in cumulus cells did not affect the formation of nuclear actin filaments in oocytes. Notably, we also observed that the nuclear actin filaments in CEOs could be induced by inhibition of gap junctions. From our results, it was confirmed that DNA DSBs induce the nuclear actin filament formation in oocyte and which is controlled by the cumulus cells.

## Introduction

The oocytes of mammalian females are arrested at the diplotene stage of first meiosis before or after their birth [1, 2]. The arrested oocyte, with its surrounding granulosa cells, forms the primordial follicle. This follicle may last for several months in case of mice and up to 10 years in humans [3]. By puberty, a part of the primordial follicles are activated and the granulosa-enclosed oocytes begin to grow. The follicle development takes about 3 weeks in mice and more than 10 months in humans [4]. When the follicles developed to the large antral follicle stage, the cumulus cell enclosed oocytes (CEOs) grow to the maximum size [5]. Under the stimulation of luteinizing hormone (LH), oocytes resume their first meiosis and are released from the ovary [6]. As a gamete, the genome integrity is essential for oocyte pass its genome to the newly formd embryo, however, some recent evidences had shown that denuded oocytes are less sensitive to the DNA double-strand breaks (DSBs) during the first meiosis resumption [7–9].

In somatic cells, DNA DSBs are firstly sensed by the Mre11/Rad50/Nbs1 (MRN) complex, and the MRN complex recruit and activate the ATM enzyme [10]. The activated ATM further phosphorylates the Serine139 of histone H2A.X to γH2A.X, which formed foci at the DSBs sites [11]. In addition, ATM also phosphorylates the DNA damage checkpoint protein Chek2 and pChek2 blocks the cell cycle and activates a serious of DNA repaired proteins [12]. If somatic cell DNA damage could not be repaired or be correctly repaired, it can lead to large scope aberrations in the genome and result in cell cycle arrest or the cell apoptosis [13]. However, for denuded oocytes (DO), slight DSBs are unable to activate the DNA damage repair pathway, and the oocytes with partial DSBs could be fertilized when the oocyte developed to the metaphase of the second meiosis [14–16]. In our previous study, we found the DNA damage response of oocytes was regulated by their surrounding cumulus cells, however, the mechanism details still haven’t been deep investigated [17].

In the cytoplasm, actin filaments mainly function as cytoskeleton to maintain cell shape and support cargo transportation. However, actin is also present in the nucleus, it plays more important roles that has been associated with many processes including gene expression, the regulation of transcription and nucleo-cytoplasmic trafficking [8, 11, 18]. Recently, a study has shown that DNA DSBs induced actin filament formation in the nucleus, which is essential for DNA damage repair [19]. For oocyte, cytoplasmic actin filaments serve as one of the main cytoskeletal systems, they could regulate various events during oocyte meiotic maturation and polarity [13]. By now, there is still no evidences showed whether the actin filament exist in the nucleus of DNA damaged oocytes. The primary focus of the present study is to analyze the nuclear actin filaments in oocyte nucleus and their association with the DNA DSBs.

## Materials and Methods

### Ethics statement

This study has been approved by the Animal Care and Ethics Committee of Qingdao Agricultural University. All animal manipulations were carried out according to the guidelines of the Animal Care and Ethics Committee. About 5–8-weeks old ICR strain mice were purchased from the Wenbo experimental animal breeding center at Qingdao city. Cervical dislocation method was used for mouse euthanasia.

### Isolation and culture of oocytes and cumulus cells

CEOs were isolated from ovaries of female ICR outbred mice 48 h post pregnant mare serum gonadotropin (PMSG) (10 IU) injection. DOs were isolated from ovaries of female ICR mice without PMSG injection. All oocyte manipulations were carried out in M2 medium (Sigma Aldrich, USA). For all the experiments, we used 2.5 μM milrinone (AmyJet Scientific, China) to block the CEOs or DOs at the germinal vesicle (GV) stage. Cumulus cells removed from the cumulus-oocyte complex (COCs) were cultured in the DMEM (Sangon) medium with 10% FBS (Sangon).

### Treatment of oocytes with drugs

In our experiments, we treated CEOs and DOs with 10 μM or 40 μM bleomycin (Selleck) to induce DNA DSBs [14]. We used the checkpoint kinase inhibitor, AZD7762 (10 nM; MedChemExpress, China), to inhibit the activity of DNA-damage checkpoint protein, Chek1/2 [20]. In addition, for blocking the gap-junctions in COC, 200 μM carbenoxolone (CBX; Sigma Aldrich, USA) was used.

### RNA-interference (RNAi)-mediated knock-down of *Adcy1*

The sequence of siRNAs used to knock-down *Adcy1* in cumulus cells was 5′- GCUGGUGAAACUGCUCAAUTT-3′; and the sequence of siRNA used as negative control was 5′- UUCUCCGAACGUGUCACGUTT-3′. The COCs were blocked in milrinone and cultured in 24-well plates. Lipofectamine 2000 (Thermo Fisher Scientific, USA) was used to transfect the siRNAs into cumulus cells, according to the manufacturer’s instructions. After 48 h, the COCs were either used for qRT-PCR or treated with BLM. To assess the rate of RNAi through qRT-PCR, total mRNA extraction was carried out from COCs, using the RNeasy Micro Kit (Qiagen Inc, Germany), followed by cDNA synthesis using TansScript One-step gRNA Removal and cDNA Synthesis Supermix (TransGen Biotech, China). qRT-PCR was performed on the LightCycler 480 II system (Roche Biotech, Switzerland), using SGExcel UltraSYBR mixture (Sangon Biotech, China). The primers used for qRT-PCR were: *Gapdh* forward, 5′-GTCATTGAGAGCAATGCCAG-3′; *Gapdh* reverse, 5′-GTGTTCCTACCCCCAATGTG-3′; *Adcy1* forward, 5′-GTGGTGGCTGCCTCGCACTT-3′; and *Adcy1* reverse, 5′-AGCAGGGCATTGGCACCGAG-3′.

### Immunostaining

To label the antibodies in oocytes, COCs and DOs were fixed in paraformaldehyde [4% solution in phosphate-buffered saline (PBS)] for 40 min, followed by permeabilization using Triton X-100 (0.1% solution in PBS) for 20–40 min. Thereafter, the oocytes were blocked with PBS containing 1% BSA for 1 h at room temperature. This was followed by incubation of oocytes with γH2A.X polyclonal antibody (1:500, Bioworld Technology, USA) overnight at 4°C. Next, the oocytes were washed with PBST buffer 3 times and incubated with secondary antibody (Anti-Rabbit IgG FITC polyclonal antibody; Sigma Aldrich, USA). This was followed by washing with PBST five times. To label the actin, oocytes were stained with tetramethylrhodamine-conjugated Phalloidin stain (AAT Bioquest, USA) for 20 min, and washed five times with PBST. Hoechst stain was used to label the nuclear DNA. Finally, the oocytes were mounted on slides and examined under a confocal laser scanning microscope (Leica, TCS SP5, Leica Microsystems).

### Western blot

The cultured mouse cumulus cell were sufficiently dissolved in 30 μL of RIPA buffer and incubated on the ice for 30 min, then added into 10 μL of 5 x SDS-PAGE (Beyotime) to centrifuge for 5 min and heated boiled water for 5 min. After that the electrophoresis, the protein were transferred to a PVDF membrane using the wet blotting system. Then the membrane was washed 3 times in TBST and blocked in 5% BSA at 4°C for 4 hours. Next, the membrane was washed in TBST for three times and incubated the Adcy1 antibody (Sigma) with dilution 1:500 at 4°C overnight. The membrane was washed in TBST for three times and the incubation of secondary antibody at room temperature for 2 h. After washing 3 times, the membranes were scanned with FluorChem FC3 and analyzed with AlphaView SA software (ProteinSimple, USA).

### Statistical analysis

Student’s *t*-test and Fisher’s exact test were performed for assessment of significance of differences. Significant differences (0.01 < p < 0.05) were marked with single asterisk (*), and extremely significant differences (p < 0.01) were marked with double asterisks (**).

## Results

### DNA DSBs induced formation of actin filaments in oocyte nucleus

**Fig 1:**
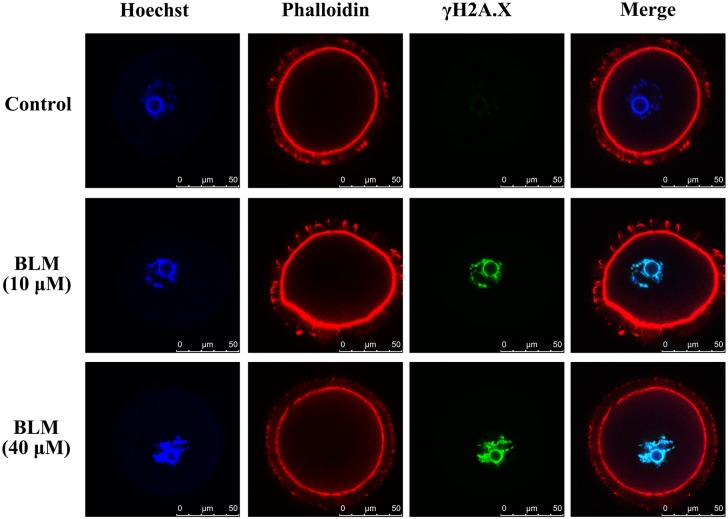
Nuclear actin filaments are not formed in the DNA-damaged DOs. The DNA DSBs (marked by γH2A.X; green) were induced by 10 μM or 40 μM BLM. The actin filaments were labeled with phalloidin.

**Fig 2:**
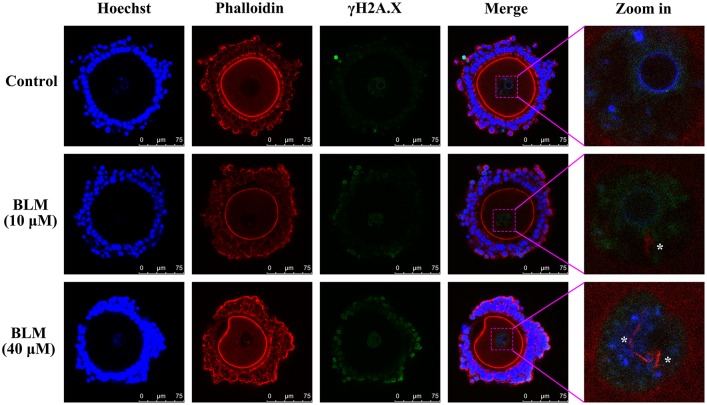
Formation of nuclear actin filaments in DNA-damaged CEOs. Treatment of CEOs with BLM for 1 h led to actin filament formation in oocyte nuclei (marked by white asterisks in the zoom-in images from last panel).

**Fig 3:**
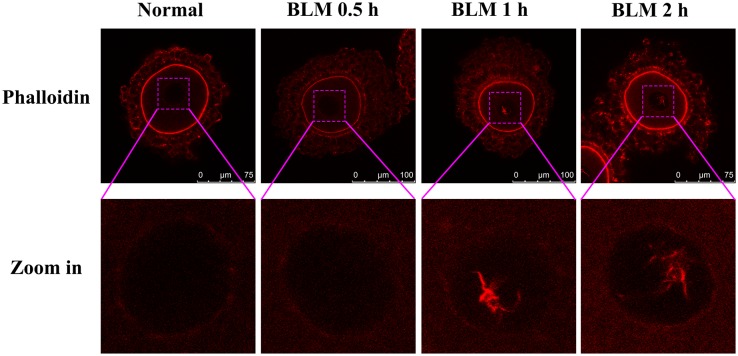
Nuclear actin filament formation upon treatment of CEOs with BLM for different time points. The nuclear actin filaments could be detected in the CEOs that were treated with BLM for 1 h or 2 h, but not in the ones treated with BLM for 0.5 h.

DOs were treated with or without BLM (10 μM and 40 μM) for 1 h to induce DNA DSBs. Then the actin filaments in oocytes were labeled with phalloidin. As a result, actin filaments were not observed in the GVs of both control oocytes and the DNA damaged oocytes (Fig 1). However, phalloidin staining of the COCs upon induction of DNA DSBs by the same method revealed the formation of nuclear actin filaments in GVs of DNA damaged CEOs (Fig 2). The results obtained also showed that with an increase in the extent of DNA damage, there was an increase in the number and length of nuclear actin filaments (Fig 2). When COCs were treated with BLM for varying time durations, nuclear actin filament formation was detected in the 1h- and 2h-treated groups, but not in the 0.5h-treated group, indicating that the nuclear actin filaments do not form immediately after the induction of DNA DSBs (Fig 3).

**Fig 4:**
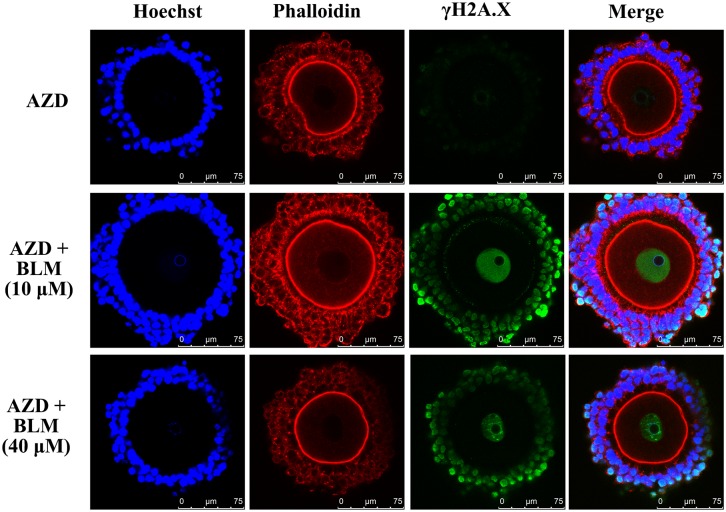
Inhibition of nuclear actin filament formation by Chek1/2 inhibitor, AZD7762 (AZD). No nuclear actin filament formation was observed upon treatment of CEOs with AZD alone. Treatment of CEOs with both BLM and AZD didn’t induce nuclear actin filament formation in oocyte nucleus.

Subsequently, AZD7762 was used to inhibit the activity of Chek1/2 protein, a conductor of the DNA damage signals [12]. When the COCs were treated with BLM and AZD7762, it was observed that AZD7762 inhibited the nuclear actin filament formation induced by the DNA DSBs (Fig 4).

### Maintenance of nuclear actin filaments in oocytes relied on the cumulus cells

**Fig 5:**
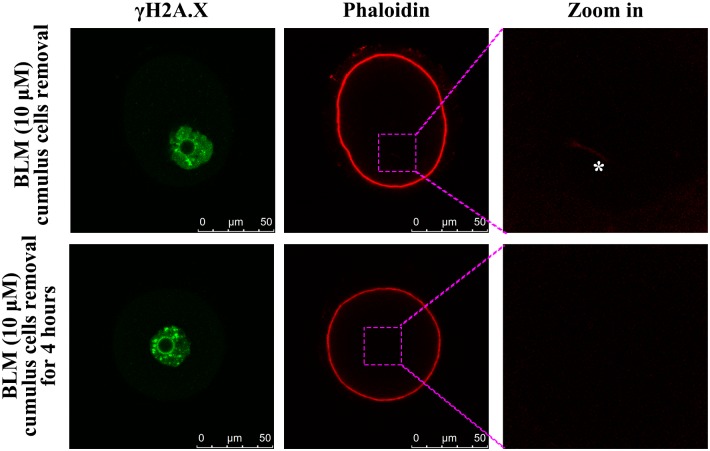
Effect of removal of cumulus cells on nuclear actin filament formation. When the cumulus cells of DNA-damaged COCs were removed and actin was stained immediately, nuclear actin filament formation (marked by white asterisk in zoom-in image in last panel) was detected. However, when the cumulus cells were removed after 4 h of incubation, majority of the nuclear actin filaments disappeared.

To analyze whether cumulus cells are essential for nuclear actin filament formation, the cumulus cells were removed from the DNA-damaged COCs (induced by 10 μM BLM), followed by labeling of actin in DOs either immediately or after 4 h of incubation. A significant reduction in the number of nuclear actin filaments was obtained when the cumulus cells were removed after 4 h of incubation, with nuclear actin filaments found only in 4 out of 16 oocytes. However, in case of the DOs, in which actin was labeled immediately after the cumulus cells were removed, the number of nuclear actin filaments was found in 13 out of 16 oocytes (p < 0.01 by Fisher’s exact test) (Fig 5).

### *Adcy1* knock-down in cumulus cells did not affect the nuclear actin filament formation

**Fig 6:**
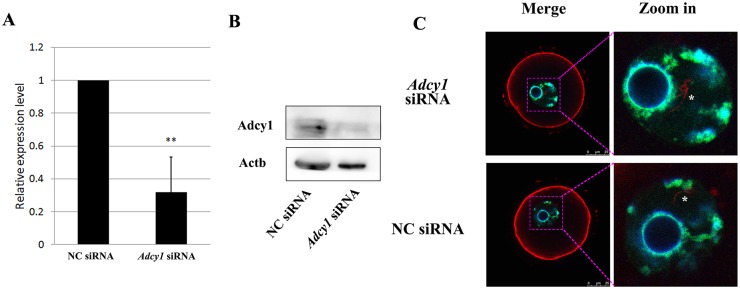
Effect of knock—down of *Adcy1* in cumulus cells on nuclear actin filament formation. (A) Efficiency of RNAi by *Adcy1* siRNA. (B) *Adcy1* siRNA transfection induced the decreasing of Adcy1 protein in cultured cumulus cells. (C) Upon knock-down of *Adcy1* mRNAs, there was no obvious change in the nuclear actin filament formation.

In our previous study, we have demonstrated earlier that the DNA DSBs led to up-regulation of *Adcy1* in cumulus cells [17]. To analyze the effect of *Adcy1* up-regulation on oocyte nuclear actin filament formation, RNAi-mediated knock-down of *Adcy1* expression in cumulus cells was carried out. Transfection of *Adcy1* siRNAs led to about 68% reduction in *Adcy1* mRNA expression as compared to the control group (Fig 6A). To confirm the RNAi efficiency of Adcy1 knocking-down in cumuclus cells, we transfected the *Adcy1* siRNAs and NC siRNAs into the cultured cumulus cells, and we found the Adcy1 protein level was decreased obviously by the RNAi experiment (Fig 6B). However, despite the down-regulation of *Adcy1* in cumulus cells, there was no significant change in the nuclear actin filaments in the DNA-damaged CEOs (Fig 6C).

### Relationship between gap junctions and oocyte nuclear actin filaments

**Fig 7:**
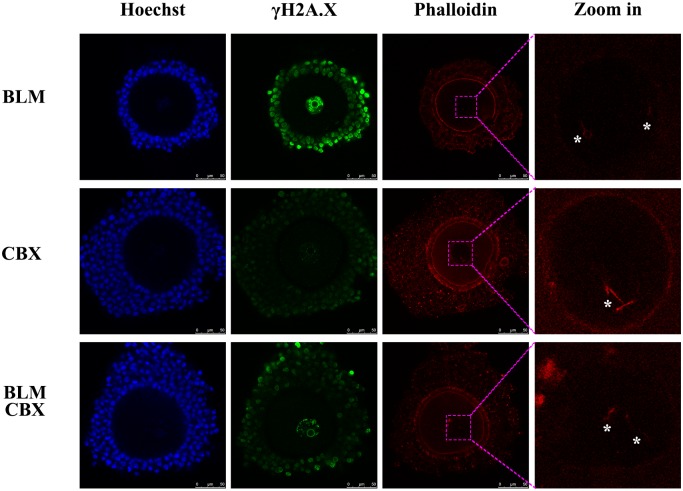
Blocking of gap junctions could induce nuclear actin filament formation in CEOs. When the CEOs were treated with the gap-junction inhibitor carbenoxolone (CBX), nuclear actin filaments formation was observed in oocytes. Corresponding to this, BLM-induced nuclear actin filaments were not inhibited by CBX.

To analyze whether the cumulus cells control oocyte nuclear actin filament formation through gap junctions, oocytes were treated with CBX to block the gap junctions between oocytes and cumulus cells. Notably, it was observed that the nuclear actin filaments were formed in the CBX-treated CEOs, even though there were no DNA DSBs in oocytes or cumulus cells. Upon simultaneous treatment of the CEOs with BLM and CBX, the actin filaments were also observed in the oocyte nucleus (Fig 7). From these results, it was confirmed that the nuclear actin filaments can be formed in the CEOs without BLM-induced DNA DSBs. This explains why CBX could not block the BLM-induced nuclear actin filament formation in CEOs.

## Discussion

Our results showed the actin filaments can form in the oocyte nucleus, but the oocyte is not able to regulate the nuclear actin filament formation by itself, and the formation and maintenance of nuclear actin filaments in the oocyte relies on cumulus cells. As the DNA damage checkpoint inhibitor could suppress the formation of actin filaments in DNA damaged CEOs, it indicated that the oocyte nuclear actin filament formation is regulated by the cumulus cells as a downstream event of DNA damage checkpoint. Our results also showed *Adcy1* knock-down in cumulus cells did not affect the nuclear actin filament formation in the DNA-damaged CEOs, which suggests the Adcy1 protein level in cumulus cells has no obvious effects on the nuclear filament actin formation. Interesting, we found blocking of gap junctions could induce the nuclear actin filament formation in the DNA-damaged CEOs, indicating the oocyte cumulus communication may involved in the nuclear actin filaments formation in oocytes.

Our previous data showed the meiosis resumption of DOs are less sensitive to DNA DSBs but DNA damaged CEOs were mostly blocked at the GV stage [17]. From this new study, we revealed the different response of DO and CEO on the DNA damage. As the nuclear actin filaments take roles on the DNA damage repair of cells [19], the formation of actin filaments in DNA damaged CEOs may indicate the exist of DNA repair events in CEOs but not DOs. Although the actin filaments were observed in the DNA damaged CEOs, whether they are the causes or the results of the cell cycle block of CEOs is still not well known.

## Conclusions

From the present study, it was concluded that actin filaments form in the nucleus of DNA-damaged oocytes only when the oocytes are surrounded by cumulus cells. The cumulus cells also play key role in the maintenance of nuclear actin filaments of oocytes. In addition, the study also revealed the important role played by gap junctions on the oocyte nuclear actin filament formation. Since blocking of gap junctions induces nuclear actin filament formation in normal CEOs, it is likely that there are certain factors that are transmitted through the gap junctions for inhibition of actin filament formation in oocytes. However, further analysis is required to decipher the factors that affect the nuclear actin filament formation.
